# Phenotype Ontologies and Cross-Species Analysis for Translational Research

**DOI:** 10.1371/journal.pgen.1004268

**Published:** 2014-04-03

**Authors:** Peter N. Robinson, Caleb Webber

**Affiliations:** 1 Institute for Medical Genetics and Human Genetics, Charité-Universitätsmedizin Berlin, Berlin, Germany; 2 Berlin Brandenburg Center for Regenerative Therapies (BCRT), Charité-Universitätsmedizin Berlin, Berlin, Germany; 3 Max Planck Institute for Molecular Genetics, Berlin, Germany; 4 Institute for Bioinformatics, Department of Mathematics and Computer Science, Freie Universität Berlin, Berlin, Germany; 5 MRC Functional Genomics Unit, Department of Physiology, Anatomy and Genetics, University of Oxford, Oxford, United Kingdom; The Wellcome Trust Centre for Human Genetics, University of Oxford, United Kingdom

## Abstract

The use of model organisms as tools for the investigation of human genetic variation has significantly and rapidly advanced our understanding of the aetiologies underlying hereditary traits. However, while equivalences in the DNA sequence of two species may be readily inferred through evolutionary models, the identification of equivalence in the phenotypic consequences resulting from comparable genetic variation is far from straightforward, limiting the value of the modelling paradigm. In this review, we provide an overview of the emerging statistical and computational approaches to objectively identify phenotypic equivalence between human and model organisms with examples from the vertebrate models, mouse and zebrafish. Firstly, we discuss enrichment approaches, which deem the most frequent phenotype among the orthologues of a set of genes associated with a common human phenotype as the orthologous phenotype, or phenolog, in the model species. Secondly, we introduce and discuss computational reasoning approaches to identify phenotypic equivalences made possible through the development of intra- and interspecies ontologies. Finally, we consider the particular challenges involved in modelling neuropsychiatric disorders, which illustrate many of the remaining difficulties in developing comprehensive and unequivocal interspecies phenotype mappings.

## Introduction

Given a candidate gene mutation thought to underlie a human phenotype, a question commonly asked by human geneticists investigating this candidacy is, “Does a mutation in this gene have a comparable effect in another species?” To answer this, animal models have either been made or identified that possess a genetic aetiology relevant to a human disorder. These models have proved themselves incredibly useful by (i) allowing repeated observations of pathologies germane to often-rare, human genetic disorders within an environmentally and genetically controlled background; (ii) enabling observations of early stages of a disorder that are often presymptomatic in humans; (iii) offering access to tissues not normally available from human patients; and (iv) providing a platform for therapeutic development and testing.

For many decades, the study in a model organism of the equivalent gene, or orthologue, of a gene associated with human phenotypic traits has delivered enormous gains in understanding [1]. Animal models carrying null mutations, or knock-outs, in the orthologues of human Mendelian disease genes have rapidly advanced our understanding of this particular class of genetic disorders, while directed mutagenesis techniques have similarly advanced our understanding of penetrant gain-of-function mutations. The ready-made, often-systematic availability of animals carrying a wide range of determined disruptions has enabled more resources to be focused on the analysis of the model rather than its generation, and projects such as the International Mouse Phenotyping Consortium are promising to revolutionise our understanding of the molecular basis of human disease by providing systematic and standardised analyses of the phenotypic relevance of nearly all mouse genes [2]–[8].

With the availability of ever more phenotype data from model organisms, the issue of what computational and algorithmic resources will be required to make optimal use of the data is becoming progressively more pressing. In this review, we will discuss how phenotypes can be mapped between humans and model species and provide a selective overview of successful approaches to cross-species phenotype mapping. Finally, we will focus on the area of neurobehavioral phenotypes, which is perhaps the most difficult of all classes of phenotypes to map between species and is representative of the challenges that remain for comprehensive cross-species mapping.

### What Is a Phenotype?

In biology, a widely accepted definition of phenotype is, “The observable traits of an organism.” In medical contexts, however, the word “phenotype” is more often used to refer to some deviation from normal morphology, physiology, or behaviour, and this is the definition that we will use here. Thus, physicians characterise the phenotype of their patients (although they rarely speak of it in this way) by taking a medical history or by means of a physical examination, diagnostic imaging, blood tests, psychological testing, and so on, in order to make the diagnosis [9].

In some contexts, the word “phenotype” is commonly used to refer to a disease entity. However, it is important to distinguish between diseases and phenotypic features. A disease usually has multiple phenotypic features; e.g., the disease “common cold” can have the features “sneezing,” “runny nose,” “fatigue,” and “fever.” On the other hand, a feature can occur with multiple diseases. For instance, “fever” occurs not only with the common cold, but also with hyperthyroidism, leukaemia, rheumatoid arthritis, and many other infectious and non-infectious diseases. Thus, there is a complex, many-to-many relationship between diseases and phenotypic features, which likely reflects the underlying pleiotropy of biochemical pathways and cellular networks.

### From Gene to Phenotype

**Figure 1:**
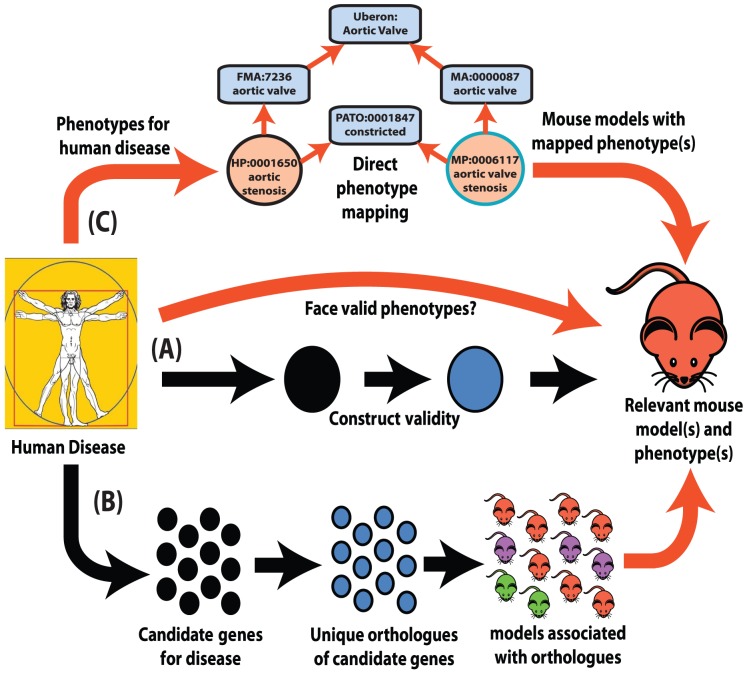
Interspecies phenotype mapping strategies. This review highlights three major methodologies to identify phenotypes in the mouse that are relevant to a human disease. (**A**) Classical approach. A mouse model is made or identified that possesses a genotype equivalent to a penetrant mutation that in human underlies the disease of interest (termed construct validity). The mouse model is examined for phenotypes that resemble those that define the human disorder (face validity). (**B**) Phenolog mapping. A group is formed containing candidate genes for a disease of interest. The respective mouse models for the orthologues of these genes are then examined for any unusually overrepresented phenotypes among them and these phenotypes (termed phenologs) are deemed relevant to the disease. (**C**) Direct phenotype mapping. Given the phenotype(s) that describe a human disease, the corresponding phenotypes in mouse are inferred by means of computational reasoning using interspecies phenotype ontology analysis. In the example shown, the HPO term *Aortic stenosis* is defined on the basis of the PATO term *constricted* and *aortic valve* (term from the Foundational Model of Anatomy ontology of human anatomy [35]). Similarly, the MPO term *aortic valve stenosis* is defined using the same PATO term *constricted* and *aortic valve* (term from the Mouse Anatomy ontology [77]). Since both the Mouse Anatomy and FMA terms for aortic valve are children of the cross-species anatomy ontology (Uberon [40]) term for aortic valve, automatic reasoning places the HPO term Aortic stenosis and the MPO term aortic valve stenosis in the direct vicinity of one another in a cross-species phenotype ontology [42]. Therefore, these terms display a high semantic similarity to one another.

Perhaps the most obvious starting point for mapping phenotypes between species is to investigate animal models with a mutation in a gene that is orthologous to a human gene associated with a disease (Figure 1A). Geneticists invoke evolution to bestow a degree of universality to the function of a gene, inferring that similarity in the encoded protein sequences implies similarity in function, and that function is most likely to be conserved between unique, 1∶1 orthologous genes [10], [11]. However, the expectation that an equivalent mutation in an orthologous pair of genes will yield the same phenotype in two different species fails to acknowledge the differences that define distinct species. A phenotype is an often complex and emergent property of a biological system that is usually influenced directly and indirectly by many genes. Even for highly penetrant mutations in close and well-conserved orthologues, significant differences in outcomes have been observed; neither the disruption of *HPRT* (Lesch-Nyhan syndrome) nor mutations in *DMD* (Duchenne's muscular dystrophy) give strong phenotypes in the mouse [12], [13]. Phenotypic differences may be observed more frequently when comparing systems, for example, immunity, that are rapidly evolving and/or subject to large environmental influence, the latter obviously not well modelled through a laboratory upbringing [14]. However, to dwell on these differences would be to deliberately ignore the many more examples of animal models that have yielded considerable insight into human genetic disease. For instance, at present, 3,829 mouse models associated to human diseases are listed in the Mouse Genome Database [15] (http://www.informatics.jax.org/vocab/omim).

Despite obvious species differences, phenotypic equivalences can be objectively discovered. The orthologues of genes that function together in a particular molecular pathway often also function together in the orthologous pathway in another species even when separated by a considerable evolutionary distance [16]. As disruptions to different genes that operate within the same pathway often produce similar phenotypes [17], disruptions of the orthologues of genes that yield a given phenotype in human can plausibly be predicted to yield the equivalent phenotype in a model organism if they are disrupting the orthologous pathway (Figure 1B).

Marcotte and colleagues systematically demonstrated this equivalence by forming groups of human genes that shared a human phenotype and then asking whether there was an unusually common phenotype amongst any one group's orthologues in another species [18]. They termed these evolutionary phenotypic associations between groups of orthologous genes “phenologs.” The thousands of phenologs discoverable through this approach included over 150 identified between human and yeast, a divergence of over 1.5 billion years. Marcotte and colleagues demonstrated that this objective approach could identify non-obvious phenologs that were of significant predictive value, showing, for example, that genes associated with lovastin sensitivity in yeast, the phenolog of abnormal angiogenesis in mice, were indeed involved in vasculature formation in *Xenopus*. In a similar approach, Webber and colleagues were able to objectively map phenotypes between human and mouse by examining the genes affected by mutations in individuals with neurodevelopmental phenotypes [19], [20]. However, while the phenolog associations revealed by these approaches are often relevant, they may not be the most specific. For example, while individuals with psychosis harbour mutations that are enriched in the orthologues of genes associated with the phenotype *abnormal prepulse inhibition* in the mouse, an abnormal prepulse inhibition is not synonymous with psychosis in humans [21]. The many-to-many relationship between genes and phenotypes makes the process of reliably mapping human phenotypes through phenologs vulnerable to pleiotropic effects and genetic interactions [22].

Perhaps the greatest difficulty in comprehensively mapping phenotypes lies in the necessary assumption that genes whose function is not associated with a particular phenotype have been examined and found not to influence that phenotype: this assumption is prolifically untrue, with only a fraction of possible phenotypes examined for only a minority of non-randomly selected genes, particularly in species such as the mouse and zebrafish that are less amenable to large-scale screening. To address this, it would be particularly valuable to identify putative phenologs in organisms amenable to high-throughput screening and thereby obtain systematic coverage (see Box 1). However, the ability to identify equivalent phenotypes between different species allows one to use genotype-phenotype associations discovered in one species to infer unexamined associations in another.

**Box 1. Comparing Phenotypes with More Distant Species**

This review has concentrated on interspecies ontology analysis of mouse, the model organism with the highest number of genes orthologous to human and the highest number of explicit models for distinct human diseases. However, while mouse models of disease often appear to most resemble their human counterparts, other model organisms offer important advantages for studying specific areas of physiology and disease-related biology. For example, the zebrafish is particularly amenable to understanding early development, due to the externally developing, transparent embryos and ease of molecular perturbation. Large-scale screens of the fruit fly *Drosophila melanogaster*, the zebrafish *Danio rerio*, and the nematode worm *Caenorhabditis elegans* mutants have been performed for several decades, and a large and diverse amount of phenotypic information has been collected. These data, while inclined towards the specific beneficial features of each model system, are complementary to one another and to mouse in their scope. Furthermore, unlike with mouse, they tend not to be as biased towards the investigation of a specific disease, as is often the case for mouse studies. The nematode worm *C. elegans* is used as a model to study cellular differentiation and basic biological processes, with the developmental fate of each of its up-to1,031 somatic cells having been mapped. The relative ease of genetic manipulation in *C. elegans* by techniques such as RNA interference [80] has enabled large-scale and largely unbiased investigations of the phenotypic consequences of alterations of gene function, and over 420,000 Worm Phenotype Ontology (WPO) annotations are available from the Wormbase [81]. Similarly, the fruit fly *Drosophila* is one of the most widely used model organisms in genetics since Thomas Hunt Morgan's discovery of chromosomes as the carriers of genes in *D. melanogaster*. Currently, over 358,000 phenotype annotations are available in the model organism database for *Drosophila* genetics, Flybase [82].

To support annotation and analysis of these models, many model organism consortia are developing phenotype ontologies that are amenable to the kind of cross-species semantic analysis described in this review, because of their use of modular definitions that make use of existing ontologies from the Open Biological Ontology (OBO) Foundry initiative [41] as building blocks. Depending on the species in question, building block ontologies representing anatomy, pathology, gene function, embryology, biochemistry, and others are used to provide computational definitions of phenotypic abnormalities. Interoperable phenotype ontologies and annotations are thus now available for human [26], mouse [29], zebrafish [75], nematode worm *C. elegans* [81], [83], fruit fly *Drosophila* [84], rat [85], and fission yeast [86]. However, one of the issues is that the nature of the genotype-phenotype annotations in each of these sources differs. In one source, the phenotypes are linked to an allele or gene, whereas in another they might be linked to a full genotype. Furthermore, since the ontologies were constructed independently and according to different principles or focus, relating them requires some sophisticated ontological engineering techniques [40]. Interoperation of these ontologies and the genotype-phenotype annotations is a primary goal of the Monarch Initiative (monarchinitiative.org), which provides integrated data and phenotype comparison analysis resources that are available to the community. Inclusion of these diverse phenotype data bring the phenotype coverage up to approximately 80% of human genes based on orthology, which may be beneficial for the identification of rare and undiagnosed genetic disease causes. Additionally, other efforts such as the Phenotype Ontology Research Coordination Network (RCN) [87] are aiming to develop standards and best practices for accurate phenotype representations across a range of plants, vertebrates, and arthropods for evolutionary biology. In the future, it will be important to improve computational methodologies for phenotypic analysis over a large range of species to make best use of the advantages that each model organism has to offer.

### Phenotype Ontologies

**Figure 2:**
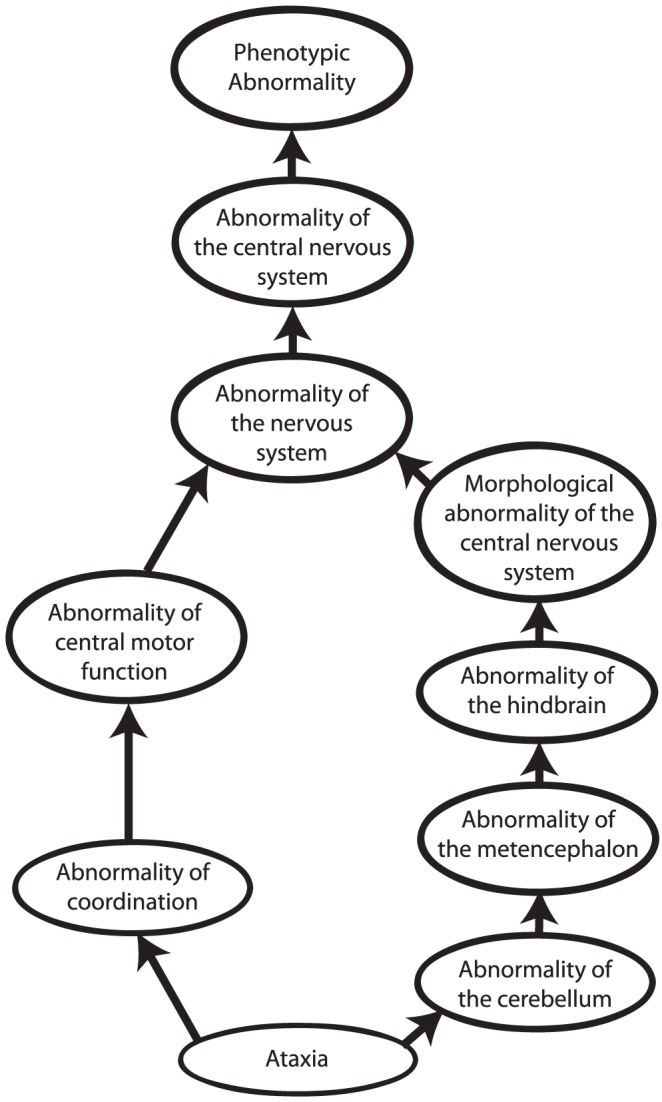
Phenotype ontologies. Phenotype ontologies (an excerpt from the Human Phenotype Ontology is shown here) consist of thousands of terms describing phenotypes arranged in a hierarchical system of subclasses and superclasses. The structure of an ontology enables annotation propagation whereby more specific phenotypic terms are also described by more general parent terms, and thus all ancestral terms. The terms are related to one another by subclass (“is a”) relations, such that the ontology can be represented as a so-called directed acyclic graph. The terms themselves do not describe any specific disease. Instead, annotations to terms are used to state that a certain disease is characterised by a certain phenotypic feature.

Other approaches to computational cross-species phenotype analysis do not begin with the identification of orthologous genes but rather directly estimate the similarity between phenotypic abnormalities seen in human disease and animal models. Ontologies have become an indispensable tool to measure cross-species phenotypic similarity. An ontology is a representation of knowledge that uses a controlled vocabulary to enable knowledge sharing and computer reasoning. “Ontology” was famously defined as a specification of a conceptualization [23], meaning that an ontology provides a representation of the concepts of a domain of knowledge (conceptualization) together with the semantic relations between them (specification). Ontologies can be used to represent items of a domain of knowledge, for example, the Chemical Entities of Biological Interest (ChEBI) ontology provides a comprehensive representation of biologically relevant small molecules [24], but also to represent the attributes of domain concepts. Perhaps the most well-known ontology of this type is the Gene Ontology [25], which describes the functions, roles, and locations of gene products. Similarly, phenotype ontologies describe the phenotypic abnormalities associated with diseases or found in individual patients or model organisms. In this review, we will concentrate on the use of the Human Phenotype Ontology [26], [27] (HPO) to describe human genetic disease, and the Mammalian Phenotype Ontology (MPO) [28], [29] to describe genetically modified mouse models [30]. Each ontology consists of thousands of terms, each of which represents a single phenotypic abnormality such as “atrial septal defect.” The terms in the HPO and MPO are related to one another by subclass (“is a”) relations, such that the ontology can be represented as a so-called “directed acyclic graph.” This structure enables annotation propagation whereby more specific phenotypic terms are also described by more general parent terms and, thus, all ancestral terms. For instance, if a patient has an *abnormality of the cerebellum*, he or she can also be said to have an *abnormality of the hindbrain*, a term that is an ancestor of *abnormality of the cerebellum* (Figure 2).

**Figure 3:**
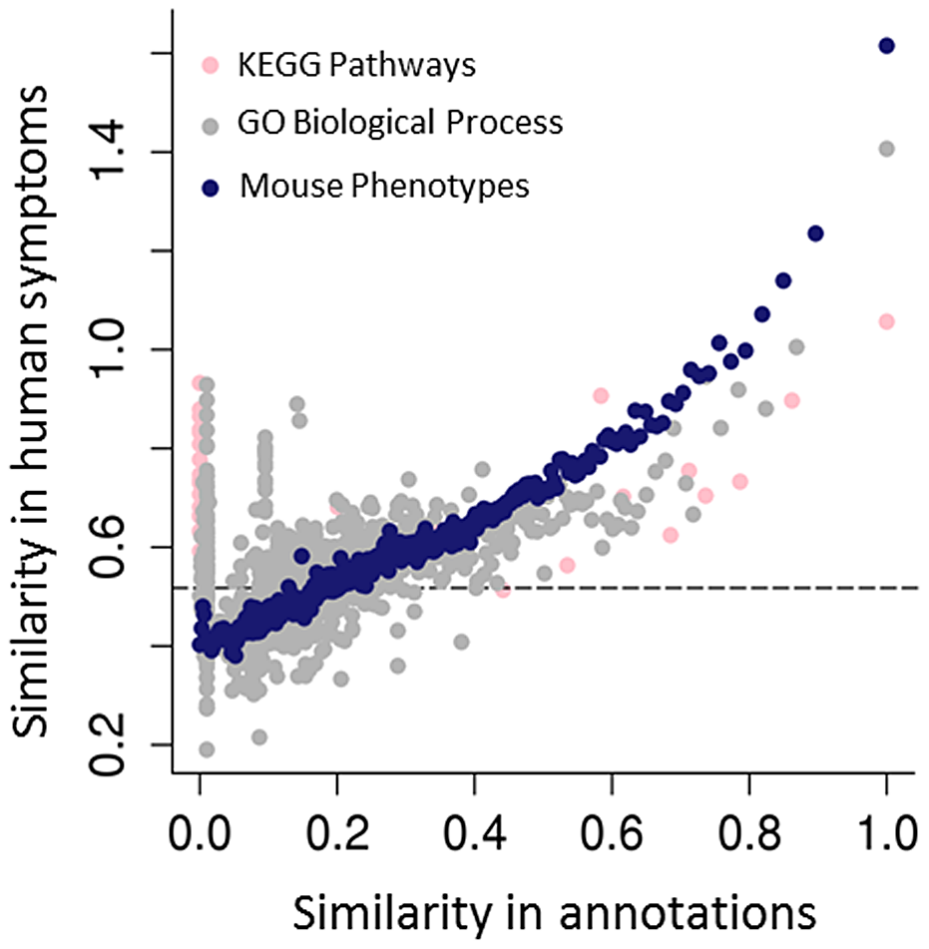
Predicting human genotype-phenotype relations from functional genomics data. The mouse phenotypes associated with the orthologues of human genes are a better predictor of genes that share human phenotypes than other popular gene annotations of the same genes, such as GO or KEGG. As both GO and KEGG include information derived from multiple sources, including annotations from the mouse, the success of the mouse phenotypes is likely due both to the genetic relevance of the mouse models and the fact that human and mouse phenotypic annotations both describe abnormalities (see Figure 1C). Resnik's [78] measure, together with the GraSM approach [79], was used to calculate the similarity of terms organised in these hierarchical ontologies, defining the semantic similarity between any two terms as the average information content of their disjunct common-ancestor terms. Gene pairs were ordered by their semantic similarity scores based on either the human KEGG pathway annotations (pink circles), human GO biological process (grey circles), or MPO annotations to genes (blue circles). For each of KEGG, GO, and MPO annotations, gene pairs were ordered in decreasing annotation similarity and grouped into bins of 2,000, and then the median semantic similarity score between gene pairs' Human Phenotype Ontology annotations was calculated. The dashed line marks the degree of similarity expected from pairs of random genes.

The phenotypic terms themselves do not describe any specific disease but may be used to list the phenotypic features that characterise a particular disease. For instance, to assert that patients with neurofibromatosis type I have Lisch nodules of the iris, we annotate the disease neurofibromatosis type I with the corresponding HPO term, “Lisch Nodules.” Mouse models that display a given phenotypic abnormality are annotated to MPO terms in an analogous fashion. The network of diseases, associated phenotypic features, and genes can now be used for a number of purposes, including differential diagnostics, prioritization of candidate genes, and research into the relationships between genotype and phenotype (Figures 1 and 3).

For some applications, it is sufficient to assert that a given genetically modified mouse is a good model of some human disease. For instance, mouse genotypes that have been used to study specific human diseases are curated by the Mouse Genome Informatics (MGI) group using disease terms found in the Online Mendelian Inheritance in Man (OMIM) [31]. While this is useful to find models for a specific disease, when starting with a set of observed phenotypes the relations described in the HPO or MPO allow us to identify all diseases (or models) characterised by those phenotypic features. Similarly, by identifying equivalent phenotypes between the HPO and the MPO, a search would be able to return both relevant mouse models and relevant human diseases. In addition to phenolog mapping described above, another approach to creating phenotypic equivalences might be to manually assign phenotype terms from one ontology to the other. For instance, one could assert that the MPO term *hypoglycemia* (MP:0000189) is equivalent to the HPO term *Hypoglycemia* (HP:0001943). While this particular mapping seems perfectly reasonable, it is not possible to map every individual term in one ontology to the equivalent term in the other ontology; many individual phenotypic features do not have a clear match in the other species, and the way a phenotype is observed and recorded in mice is often quite distinct from phenotypic analysis performed in the course of a medical examination. For instance, there is no obvious match in humans for the MPO term *abnormal tail movements* (MP:0001391), and there is no obvious match in mice for the HPO term *Expressive language delay* (HP: 0002474). Another important issue is that phenotypes elicited in the course of scientific experiments on mouse models are not equivalent to medical phenotypes. For instance, it is not uncommon to subject mouse hearts to ischemia and reperfusion to induce cardiac damage, and then to compare the hearts of mice with a certain genetic defect to those of wild-type mice. If the mutant mice exhibit larger infarctions than the wild-type mice, the MPO term *increased myocardial infarction size* (MP:0003037) is used to annotate them. Obviously, there is no corresponding HPO term, and in fact it is not even entirely clear what the relationship of *increased myocardial infarction size* to the HPO term *Myocardial infarction* should be.

For this reason, a different strategy was chosen to develop semantic mappings between the HPO and the MPO. A crucial part of this strategy is the use of *logical definitions* to enable sophisticated semantic reasoning over ontology terms. Logical definitions of phenotype terms use building block ontologies to represent the various anatomical, cellular, physiological, and metabolic abnormalities, combining them into ontology classes using semantic constructs of the ontology language OWL. The Phenotype, Attribute, and Trait Ontology (PATO) is a key tool in this effort because it provides an abstract representation of the abnormal qualities encountered in the phenotypic abnormalities (Figure 1C) [32]–[34]. PATO consists of a single hierarchy of qualities designed to be used in conjunction with other ontologies representing entities that are the bearers of abnormal phenotypic qualities, including the Foundational Model of Anatomy (FMA) ontology [35], the Gene Ontology (GO) [36], and the cell ontology [37]. Many phenotype terms can be defined using the Entity/Quality paradigm. In the following example, we consider an HPO term that describes increased width of the big toe.

Class: HP:0010055
Annotations: label “Broad hallux”
EquivalentClassOf:
**has_part some**:
increased width (PATO_0000600) **and**
**inheres_in some** Big toe FMA:25047

That is, the HPO class “Broad hallux” is defined as being equivalent to a phenotype of *increased width* that inheres in (is located in) a *Big toe*. Many phenotype terms require more complicated definitions that include references to multiple domain ontologies. For instance, the following definition of the HPO term *Hyperalaninemia* uses references both to the FMA term for blood and to ChEBI for alanine.

**Class:** HP: 0003348
**Annotations: label** “Hyperalaninemia”
**EquivalentClassOf:**
**has_part some:**
increased concentration (PATO:0001162) **and**
**inheres_in** Portion of blood (FMA:9670) **and**
**towards** alanine (CHEBI:16449)

These definitions enable interoperability of the HPO with the other ontologies in the sense that it becomes possible to search for all phenotype terms that involve entities from one of the domain ontologies, comprising not only anatomy and small molecules as shown above, but also gene function [36], cell types [37], proteins [38], pathology [39], and others. Also, thanks to Uberon, an integrated cross-species ontology, it is possible to map anatomical terms across species [40]. Thus, by using logical definitions for mouse and human phenotypes that have been developed using interoperable ontologies from the Open Biomedical Ontology (OBO) Foundry [41], a common computational basis is created that in turn makes it possible to identify equivalent or similar terms between phenotype ontologies for different species by using automatic reasoning [42]–[44].

A number of different approaches to interspecies phenotype mapping have been applied by several groups, and we will refer to the original publications for algorithmic details [32], [43], [45]–[47]. However, for the most part the algorithms make use of logical definitions as shown above to identify equivalencies or similarities between terms of phenotype ontologies for two or more species (Figure 1C). Each animal disease model or human disease is then annotated to one or more ontology terms. For instance, the human disease Marfan syndrome is annotated to a number of HPO terms including *Tall stature* (HP:0000098), *Kyphoscoliosis* (HP:0002751), *Ectopia lentis* (HP:0001083), and *Aortic root dilatation* (HP:0002616). The mgR mouse model of Marfan syndrome [48] is annotated to a number of MPO terms including *increased length of long bones* (MP:0004695), which is similar to the HPO term *Tall stature* (which in Marfan syndrome results from overgrowth of the long bones); *kyphosis* (MP:0000160), which is similar to the HPO term *Kyphoscoliosis*; and *aortic aneurysm* (MP:0006278), which is similar to the HPO term *Aortic root dilatation* (an aneurysm is a protruding sac formed by the dilation of the wall of the aorta, whereas the term *Aortic root dilatation* refers to an increase in the diameter of the proximal section of the aorta). The mgR model does not display an ocular phenotype, so there is no obvious match for the HPO term *Ectopia lentis* (a dislocation of the lens of the eye). To calculate a phenotypic similarity between the human disease and the mouse model, the algorithms mentioned above search over each of the terms of the human disease and look for the best match amongst the terms used to annotate the mouse model and vice versa. The sum of the similarities, which are usually expressed using their information content, is then used as a measure of the similarity between the diseases. The information content is calculated based on the frequency with which a given ontology term is used to annotate diseases in a database and thereby provides a way of weighting the matches based on the specificity of the phenotypic features: the less specific a phenotype is, the lower the information content. Many algorithms have been presented to calculate this kind of semantic similarity with ontologies, and the field represents an area of active research in bioinformatics [49]. Another recent approach to the use of ontologies for differential diagnostics in human medicine did not rely on semantic similarity algorithms but rather embedded the HPO and the diseases annotated to terms of the HPO into a Bayesian network, thereby providing a principled framework to deal with noise in phenotypic data and demonstrating a substantially improved performance on simulated data [50]. Although the field of semantic phenotype matching is still in its infancy, even now mouse data are demonstrably better at identifying genes that influence the same human phenotypes than other commonly used gene annotations such as Gene Ontology or Kyoto Encyclopaedia of Genes and Genomes (KEGG) (Figure 3). Robinson and colleagues have also recently shown that cross-species phenotype matching is a powerful method for the prioritization of candidate genes in whole-exome sequencing studies [51].

## Cross-Species Analysis of Behavioural Phenotypes and Elucidating the Genetic Architecture of Psychiatric Disease

Behavioural disorders, notably psychiatric disorders, present a particularly difficult challenge to both phenotype ontologies and cross-species analysis. For example, determining the presence in a mouse model of any of the new Diagnostic and Statistical Manual of Mental Disorders, Fifth Edition (DSM V) positive symptoms required in the diagnosis of schizophrenia (“hallucinations,” “delusions,” or “disorganised speech”) is clearly problematic [52]. Furthermore, current psychiatric diagnostic classifications similarly label patients presenting with a broad spectrum of phenotypes, and heterogeneous presentations likely result from heterogeneous aetiologies. However, given a well-characterised and large cohort of patients harbouring likely highly penetrant mutations, relevant mouse model phenotypes can still be objectively discovered: considering genes affected by *de novo* copy-number variations in 186 individuals with autism, Webber and colleagues were able to associate over 40 phenologs which were well correlated to the phenotypes already observed in existing mouse models of autism-associated genes [53]. However, while the association identified between autism and the mouse phenotype s*tereotypic behaviour* is readily comparable to the autistic phenotype of *repetitive behaviours and interests*, there were no clear and specific associations to the impaired social interaction and verbal and non-verbal communication deficits that also define autism [54].

Results of genome-wide association studies (GWAS) suggest that current approaches to the diagnosis and classification of psychiatric diseases are inadequate. For instance, GWAS findings have challenged the traditional distinction between schizophrenia and bipolar disorder by identifying genes such as *CACNA1C* that harbour risk alleles for both disorders [55]. Such findings did not, perhaps, come as a complete surprise given the fact that relatives of probands with either disorder have increased risks of both schizophrenia and bipolar disorder [56], as well as the well-known clinical overlap between the two: patients with bipolar disorder can have episodes of psychosis during either manic or depressed phases. In fact, more recent findings show that specific single nucleotide polymorphisms can be associated with a range of psychiatric disorders of childhood or adult onset [57].

We join with others to suggest that it may be beneficial to take a new approach to the analysis of neurobehavioural disorders that will focus on the individual components of the disorder rather than just the final diagnostic category [58]. This new approach has two potential benefits. While the main clinical purpose of a diagnostic category is to allow therapeutic and prognostic decision making, it is arguable that the most useful clinical categories, phenotypic features, dimensional definitions, and measures for psychiatric disease are still unknown [55]. Therefore, the act of reducing probably heterogeneous groups of patients to a single clinical category such as bipolar disorder is likely to reduce the power of GWAS or sequencing studies to elucidate the molecular pathology of psychiatric disease. Studies based on richer representations of the phenotype may, in contrast, allow new hypotheses to be tested, such as that a certain genetic variant is a risk factor for psychosis, rather than schizophrenia or bipolar disorder per se [59].

The second potential benefit of this approach for neurobehavioral clinical research is an improved ability to make use of animal models to understand psychiatric disease by allowing more accurate interspecies phenotypic comparisons on the basis of individual phenotypic aspects of a disorder rather than on complex emergent phenomena associated with a disorder. For instance, glucocorticoids influence neuronal function in the brain, and are thought to be involved in the onset of depression when levels are abnormally high [60]. However, it is still unclear how glucocorticoid signalling is linked to affective disorders. A zebrafish mutant with a mutation in the glucocorticoid receptor was shown to become immobile (“freeze”) and to show reduced exploratory behaviour when placed into an unfamiliar aquarium (“novel tank”), abnormalities that could be reversed by the addition of the antidepressant fluoxetine to the holding water [61]. While it appears quite reasonable to infer that this zebrafish is modelling some aspect of depressive psychopathology, it is presumably not a faithful model of any specific human disorder, such as major depressive disorder, with symptoms such as feelings of excessive or inappropriate guilt or suicidal ideation.

The above considerations fit well with the so-called endophenotype concept in psychiatric genetics. An endophenotype in psychiatry refers to an internal process that can be objectively measured. An endophenotype is an individual feature that may be a component of a psychiatric disease. Psychiatric endophenotypes are defined as being heritable features that tend to manifest in individuals with psychiatric diseases whether or not the disease itself is active, and that not only cosegregate in families together with the disease but also tend to be found in unaffected relatives of an individual with a psychiatric disease at a higher rate than in the general population [62]. One main reason why endophenotypes have attracted attention is the assumption that if an endophenotype represents a more or less atomic component of a complex disease entity, then the number of genes required to produce variations in these traits may be fewer than those involved in producing a psychiatric diagnostic entity, making it easier to identify genetic factors for endophenotypes than for disease entities [63]. Although a meta-analysis published in 2007, i.e., before the publication of large-scale psychiatric GWAS, failed to show an advantage for the analysis of endophenotypes in the identification of risk alleles for schizophrenia [62], more recent results have identified loci significantly associated with various endophenotypes in schizophrenia [64], [65]. However, it should not necessarily be assumed that endophenotypes themselves have a simpler genetic architecture than psychiatric illnesses. Additionally, what appears to be an equivalent endophenotype in human and mouse may actually reflect a different pathophysiology. For instance, deficits in mouse spatial working memory have recently been reported to be based in the hippocampus, questioning the face validity of this phenotype for deficits in working memory associated with scz/bpd in humans, the latter based in the frontal cortex [66]. That said, in many cases genetically altered mice do seem to provide valid models for aspects of human psychiatric diseases. For instance, schizophrenic patients report oversensitivity to sensory stimulation that possibly could be related to the cognitive fragmentation seen in this disorder. Experiments with cortical event-related potentials and the prepulse inhibition of startle responses have shown that schizophrenic patients also have impaired central nervous system inhibition (sensorimotor gating) [67]. Correspondingly, neuregulin 1 (*NRG1*) is a schizophrenia susceptibility gene in humans, and mice lacking any one of the several isoforms of *Nrg1* display deficits in sensorimotor gating [68], among other abnormalities that resemble some of the features of human schizophrenia. Similarly, the *SNAP25* gene has been linked to schizophrenia in association studies [69], and mouse models with abnormalities in *SNAP25* have been shown to have abnormalities in rest and activity rhythms, reminiscent of the disturbed sleep patterns observed in schizophrenia [70]. Therefore, if appropriate caution is exercised in the interpretation of results, a case can be made that it may be simpler to investigate the genetic correlates of psychiatric endophenotypes in mouse models. Indeed, analogous touch-screen tests performed by humans and by mice, both carrying mutations in *DLG2*, a gene implicated in schizophrenia, have demonstrated comparable cognitive impairments, illustrating that endophenotypes can be much more directly and readily equated between species [71]. It has been proposed that one way of improving our understanding of the underlying molecular mechanisms of neurobehavioural diseases such as schizophrenia lies in the statistical cross-comparison of datasets arising from analyses of animal models and human studies, which will identify experimental and clinical biomarkers. Such findings would lend credibility to the animal models and could potentially be used to monitor treatment effects in these models [72]. To achieve this goal, we suggest that targeted work on developing comprehensive and consistent ontological representations of the neurobehavioral phenotypes in humans, mice, and zebrafish would be quite valuable.

### Conclusions

The human genome project was compared by Victor McKusick to the anatomical atlas of Vesalius published in 1543, in that both works provided for the first time a comprehensive list of parts that the human body (or genome) contains, but did not actually explain how the parts work together to mediate function. William Harvey capitalised on the knowledge contained in the Vesalius atlas to describe the basic principles of the circulation 85 years after the publication of the atlas [73]. Similarly, the challenge for the coming decades will be to assign physiological functions and medical roles to the parts of the genomic atlas and to begin to understand how the parts fit together into larger systems. Current large-scale projects, including the International Mouse Phenotyping Consortium [74] and the ever-growing amount of data being organised by resources such as the Zebrafish Model Organism Database [75], stand to play a transformative role in this effort by providing a comprehensive view of the phenotypic consequences of the majority of protein-coding genes in the vertebrate and mammalian repertoire. Similar resources are being developed for the investigation of microRNA genes [76], and it is a good bet that regulatory sequences such as tissue-specific enhancers will be next in line. Computational analysis of the phenotype will play a critical role in these efforts. In this review, we have highlighted a number of computational resources and algorithms that have been developed to address current challenges in the field, but it seems fair to say that the field of computational phenotype analysis is still in its infancy. Nonetheless, computational interspecies phenotype analysis will play a crucial role to make full use of the data emerging from large-scale projects, such as the International Mouse Phenotyping Consortium and the Zebrafish Mutation Project, that stand to translate the genomic atlas into functional and medical discoveries that will improve our ability to treat human disease.
